# Exploring the carry-over of top-down attentional settings in dynamic conditions

**DOI:** 10.1177/17470218231155018

**Published:** 2023-02-10

**Authors:** Catherine Thompson, Maryam Jalali, Peter J Hills

**Affiliations:** 1 Department of Psychology, Liverpool Hope University, Liverpool, UK; 2 School of Health and Society, University of Salford, Salford, UK; 3 Department of Psychology, Bournemouth University, Poole, UK

**Keywords:** Top-down attentional set, set switching, attentional inertia, eye movements, attentional control

## Abstract

A top-down attentional set can persist from a relevant task to an irrelevant task, influencing allocation of attentional resources, visual search, and performance. While this “carry-over” effect has been found across numerous experiments, past studies have utilised paradigms that present similar tasks to the same spatial location. The present research explored whether attentional settings persist in more dynamic situations. In Experiment 1, participants played a computer game that encouraged a horizontal, vertical, or random spread of search. After 10 or 30 s, they moved 90° to their right and monitored a driving video for hazards. Eye movements to the videos were not affected by the characteristics of the preceding game, revealing no carry-over of attentional settings. One possible explanation for this was the visuospatial shift between the tasks. To explore this further, Experiment 2 adopted a similar paradigm to previous research; participants searched horizontal, vertical, or random letter strings before completing an image search. In one block the tasks were presented to the same screen, and in one block the tasks were presented to different screens (incorporating a 90° visuospatial shift mid-trial). Carry-over was found in the one-screen block, with a significantly wider horizontal search and a narrower vertical search in the pictures after a horizontal letter search. However, there was no carry-over from the letter to the picture task in the two-screen block. This indicates the flexibility of attentional control in dynamic situations, and it is suggested that persistence of attentional settings will be most costly under stable conditions.

## Introduction

It has been proposed that the attentional orienting system is configured to selectively attend to items that are relevant to a task through the establishment of an attentional control setting (Folk et al., 1992). This is known as the top-down attentional set and has been defined as “a preparatory state of the information processing system that prioritizes stimuli for selection based on simple visual features” (Leber & Egeth, 2006, p. 565). An attentional set supports selective visual attention by biasing resources towards relevant information and away from irrelevant information (e.g., Folk et al., 1992; Johnston & Dark, 1986; Schneider & Shiffrin, 1977; Theeuwes, 1993). The set should be based on the demands of a given task, and therefore when a task changes the attentional settings should be updated.

Yet studies have shown that once a top-down set is established, it may not always be updated as the task demands change. For example, using a rapid serial visual presentation task (RSVP), Leber and Egeth (2006) found that participants allocated attention towards irrelevant distracters in a second block of trials based on the instructions given for an initial block of trials. In a “training” block, one group searched for a specific-coloured target presented in a stream of different coloured distracters (feature search) and a second group searched for a coloured target presented in a stream of grey distracters (singleton search). In a subsequent “test” block of trials, all participants had to complete a feature search, yet the singleton group showed attentional capture from the irrelevant distracters because their attention was still set to search for a coloured target, rather than a specific-coloured target. Thompson et al. (2007) found a similar effect with an RSVP task. In an initial block of trials participants had to identify two targets (a number and a vowel) from a stream of irrelevant consonants and results showed a standard attentional blink effect, whereby accuracy to Target 2 was impaired when presented in close temporal proximity to Target 1 (e.g., Raymond et al., 1992; Shapiro et al., 1997). In a second block, Target 1 (the number) was irrelevant and participants were only asked to identify the vowel, however, a significant attentional blink remained, showing that attention was still being allocated to this item. Crucially, the carry-over of attentional set had a negative impact on accuracy in both studies, showing that top-down settings are not always updated in accordance with a task change even when this change would benefit performance. Leber and Egeth (2006) proposed that when resources have been invested in the establishment and maintenance of an attentional set, it will persevere until the costs of using an inefficient set outweigh the costs of switching set.

Thompson and Crundall (2011) criticised these studies because they measured the persistence of attentional set following significant practice with an initial task and the second task incorporated the same stimuli as the first. Consequently, the experiments do not represent how attention works in the dynamic environment outside the laboratory. Lien et al. (2010) made a similar argument, suggesting that real-world behaviour requires frequent changes in attentional settings. They found limited evidence of any persistence of attentional control settings when participants were required to switch set every few seconds, and this led them to conclude that attentional control is flexible in dynamic settings. Yet while they measured the ability to switch between two sets (and how this affected the contingent capture of attention by irrelevant items that shared a previously relevant target-defining feature), the two sets were always in direct competition. Lien et al. acknowledged that persistence of attentional settings may still occur in dynamic conditions, providing that the relevant features in one task are different to the irrelevant features in the other task.

Expanding on this, Thompson and Crundall (2011) developed a paradigm in which participants were asked to complete two distinct tasks, spending a limited amount of time in each task. In every trial, an initial task required a search through nine letters to make a response regarding the number of vowels presented (either three or four). The letters were arranged in a horizontal line across the centre of the screen, a vertical line down the middle of the screen, or randomly across the screen. To raise the uncertainty of when the second task would begin, participants were presented with one-, two-, or three-letter searches in each trial. Following the letter search(es), a picture of a road was shown for 2 s and participants were asked to view the image in preparation for a memory test (Experiment 1) or rate the image for how hazardous the road was (Experiment 2). Eye movements in the picture search were then compared based on the orientation of the preceding letters. Despite a small amount of time attending to the letters, and the fact that the two tasks in each trial incorporated very different stimuli, a carry-over of attention was found, whereby vertical search in the pictures increased when they were preceded by vertically oriented letters. A third experiment found the effect extended to video clips of driving, with vertical spread of search narrower following a horizontal letter search. This shows the carry-over of top-down attentional settings between two unrelated tasks.

In line with theoretical accounts of task switching (e.g., Allport et al., 1994; Monsell, 2003; Schmitz & Voss, 2011), Longman et al. (2013) suggested that when a task changes, the previously relevant attentional settings need to be inhibited before the new attentional settings can be adopted. Until the previous settings can be inhibited, they will continue to influence the allocation of attention, an effect they term “attentional inertia.” The importance of inhibition is supported by findings showing that when the intertrial interval between two different tasks is longer (enabling more time for inhibition of the previously relevant settings), the attentional inertia effect is smaller (Longman et al., 2017). Similarly, Thompson and Crundall (2011) suggested that attentional weights (i.e., Bundesen, 1990; Bundesen et al., 2015) are assigned to relevant stimuli and spatial locations in a given task and when the task changes, these weights persist and have an impact on the allocation of attention and spread of search in the new task.

Since the investigation of Thompson and Crundall (2011), several other studies have demonstrated the carry-over of top-down attentional settings, using the same or similar paradigm (e.g., Hills et al., 2016, 2017; Thompson et al., 2015). However, it may again be argued that while this paradigm is more “dynamic,” it is still limited because the tasks are always presented to the same spatial location. In addition, while attempts have been made to change stimuli in the second task (using faces, images of real-world scenes, and videos) the initial task of searching through letters remains artificial. Consequently, it is unclear whether carry-over contributes to the allocation of attention outside a laboratory setting. The aim of the present work was to investigate whether the carry-over effect occurs under more flexible conditions. In the first experiment, participants played games on a tablet and were then asked to watch a driving video on a computer and identify any hazards. The games were chosen because they induced different visual search strategies, with one being more horizontal, one more vertical, and one more random. Eye movements in the videos were compared across these three conditions to measure whether the allocation of attention in the different games would persist to the videos.

Experiment 1 showed no carry-over effect and one explanation for this was that participants had to move (through space) between two different displays. This was investigated in a second experiment that reverted to the original paradigm of Thompson and Crundall (2011), presenting a letter search followed by a picture search; however, the two tasks were shown on the same screen or on two different screens. The aim was to investigate whether the spatial switch between the two tasks affects the carry-over of attentional set. If so, this would be illustrated by a carry-over effect when both tasks were shown to the same screen, but not when they were presented to different screens.

## Experiment 1

The aim of Experiment 1 was to explore whether the persistence of top-down attentional set would occur in more dynamic settings. Arguably, the closest the previous research has come to this is by demonstrating a carry-over of visual search from simple letter strings to hazard perception clips (Hills et al., 2018, 2021; Thompson & Crundall, 2011). This work has also shown that persistence of a more vertical visual search (following a search through vertically oriented letter strings) has a negative impact on hazard perception. Building on these past studies, Experiment 1 measured persistence of visual search to driving videos but changed the initial task from a letter search to a computer game. This was more reflective of a dynamic setting because in addition to incorporating different demands the tasks were presented to different spatial locations. Three different games were selected on the basis that they encouraged a more horizontal, vertical, or random spread of search. In each trial participants played the selected game for 10 or 30 s before moving to a second display and searching a driving clip, returning to the game when the clip ended (ready for the next trial). The amount of time spent playing the game before moving attention to the driving clip was manipulated following the findings of Thompson et al. (2015) that greater engagement with the initial task increases the carry-over effect.

While still far removed from a real-world scenario, the design of this experiment allowed an investigation of how search in a “driving” task (hazard perception clips) would be affected by the allocation of attention in a “non-driving” task (a computer game). There is reason to propose that carry-over of attentional settings can occur within the driving task due to the findings surrounding the “out of the loop” problem. When a driver allocates their attention away from the primary driving task to a non-driving task (e.g., talking, texting, programming a satellite navigation system) they are referred to as being “out of the loop” (Endsley & Kiris, 1995). Merat et al. (2014) found that once out of the loop it can take between 10 and 15 s for a driver to re-focus their attention on the road and re-gain control of the vehicle. Although the carry-over effect is relatively small in comparison with other top-down and bottom-up influences upon attentional resources in the driving task, it may be the case that persisting attentional settings could be contributing to the out of the loop problem.

It was predicted that if carry-over occurs in dynamic conditions when individuals are completing different tasks shown to different spatial locations participants would show wider horizontal spread of search following the horizontal game and wider vertical spread of search following the vertical game. In addition, the influence of the game would be greater as participants spent more time completing it before viewing a driving clip. If there was no evidence of carry-over this would suggest that persistence of attentional set will not occur in more dynamic settings.

### Method

#### Design

The experiment had a 3 (orientation) × 2 (duration) within-participants design. The *orientation* related to the game played on the tablet with the games each inducing a different spread of visual search (horizontal, vertical, or random). The *duration* was the time spent engaging in the secondary task before participants were prompted to move their attention to the driving clips (10 or 30 s). The dependent variables were spread of search along the horizontal and vertical axis in the first 1,500 ms of the driving clips (operationalised as the *SD* of the *x* and *y* fixation positions and measured in degrees of visual angle). Accuracy and response times were also recorded to the hazard perception clips. Full ethical approval was obtained from the School of Health Sciences Research Ethics Panel at the University of Salford (HSCR 15-83).

#### Participants

The sample size was determined through an a-priori power analysis using G-Power based on the effect size from a 3 (orientation) × 2 (time) interaction found by Thompson et al. (2021). Using this prior effect size of *f* = 0.32, to achieve a significant result with 0.95 power at least 33 participants were required. A total of forty participants (20 female) completed the experiment, all were staff or students from the University of Salford, and all held a valid driving licence. Age ranged from 19 to 45 with a mean age of 28.38. Participants were given an inconvenience allowance of £10 for completing the experiment. All participants reported normal or corrected-to-normal vision.

#### Stimuli and apparatus

The experimental task involved participants playing games on a tablet and searching hazard perception clips on a computer. E-Prime Pro 2 was used to present the hazard perception clips and record accuracy and response times. These were presented on a Viglen genie computer with an Intel Core i7-3770 processor and an 18.5-in. screen (aspect ratio of 4:3). The videos were presented to the full screen and had a resolution of 1,280 × 1,024 pixels. E-Prime was also used to provide a tone (ElectronicChimeSound.mp3) indicating when participants should move from the tablet game to the hazard perception clips. When viewing the hazard perception clips participants were asked to place their chin in a chin rest and were seated 60 cm from the screen. A Tobii X3-120 eye-tracker recorded eye movements to the clips. This had a sampling rate of 120 Hz. The minimum fixation duration was 100 ms and the minimum fixation dispersion threshold was 100 pixels.

Three tablet-based games were selected for the secondary task, Solitaire, Tetris, and Jewel Fever. Solitaire is a card game that involves grouping playing cards onto a series of horizontally presented piles in a set order, and then adding the cards to piles of the same suit. Participants moved the cards by touching the screen and dragging each one to the chosen location. In the game Tetris different angular shapes are presented one at a time and move from the top of the display to the bottom. The goal is to rotate each shape and move it so that it fits with other shapes in a line along the bottom of the display. As soon as a line is completed it disappears. Participants touched the screen to rotate the shapes and could drag them left, right, and downwards as required. In the Jewel Fever game, a random display of coloured shapes is presented in an 8x8 square grid and players need to match three identical coloured shapes by selecting them and moving them next to each other. Participants touched the screen to drag the shapes, and when matched the shapes disappeared and were replaced by new shapes.

**Figure 1.**
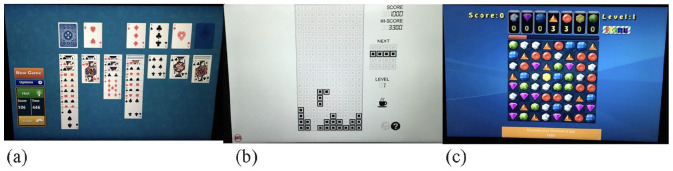
Images from the three tablet-based games chosen for the secondary task in Experiment 1. From left to right the games are Solitaire (predominantly horizontal search), Tetris (vertical search), and Jewel Fever (random search).

The games were presented on a Microsoft Pro Surface, each was presented to the full screen (12-in.) but the stimuli in each game occupied a specific area of space (see Figure 1, for example, images). To ensure that the three games elicited a different visual search they were piloted with ten participants. The participants were asked to spend 5 minutes playing each game using the Surface while their eye movements were measured with a Tobii X2-60. This had a sampling rate of 60 Hz, the minimum fixation duration was 100 ms, and the minimum fixation dispersion threshold was 100 pixels. A mobile device stand was used to hold the tablet with the eye-tracker situated below and participants were asked to position their arms outside of the bars to ensure they did not block the eye-tracker. The order of the games was randomised, and data was collected from nine participants after a calibration issue with one participant.

**Figure 2.**
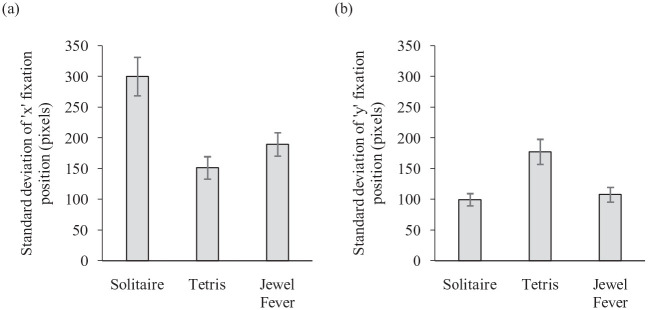
The standard deviation of the (a) *x* and (b) *y* fixation positions (measured in pixels) in the pilot experiment for the Solitaire, Tetris, and Jewel Fever games. Error bars represent standard error.

Analysis of the horizontal and vertical spread of search in the three games was conducted using the *SD* of the *x* and *y* locations of each fixation (measured in pixels). Search was compared across the three games using two within-participants ANOVAs. For horizontal spread of search there was a significant effect of game, *F*(2, 16) = 47.462, *MSE* = 1,130.610, *p* *<* .001, *partial* η^2^ = .856; see Figure 2a. Pairwise comparisons with Bonferroni corrections showed significantly wider horizontal search in the Solitaire game (*M* = 300.08 pixels) compared with both Tetris (*M* = 151.40 pixels; *p* *=* .001) and Jewel Fever (*M* = 189.59 pixels; *p* *<* .001). Horizontal search was also significantly wider in the Jewel Fever game than the Tetris game (*p* *=* .002). The effect of game was also significant for vertical spread of search, *F*(2, 16) = 38.761, *MSE* = 426.234, *p* *<* .001, *partial* η^2^ = .829; see Figure 2b. Vertical search was significantly wider for Tetris (*M* = 177.48 pixels) compared with Solitaire (*M* = 99.45 pixels; *p* *=* .001) and Jewel Fever (*M* = 107.79 pixels; *p* *<* .001). There was no significant difference in spread of search along the vertical axis for the Solitaire and Jewel Fever games (*p* *=* .501). Based on these results Solitaire was considered a “horizontal” search, Tetris a “vertical” search, and Jewel Fever a “random” search. It should be noted that search along the horizontal axis in the Tetris game was still moderate, and while it did lead to the most vertical search of the three games, there is an argument that it does not enable a clear dichotomy between a horizontal condition and a vertical condition.

#### Procedure

Participants were given full instructions for the task and provided written informed consent. They were asked to sit with their chin in the chin rest in front of the computer screen with the tablet situated on a desk to their left-hand side. Eye movements were calibrated using a 5-point calibration screen and then participants were instructed to take their chin out of the chin rest and turn 90° to their left to begin the tablet game. They spent either 10 or 30 s on the tablet task before a tone sounded on the computer for 1,000 ms indicating that they should turn 90° to the right, place their chin in the chin rest again and monitor the presented driving clip for hazards. A driving clip was shown for 15 s, and participants were asked to press the spacebar if they saw a hazard at any point during the clip. Half the videos featured a hazard and half had no hazard. For clips that did include a hazard this could only occur in the final 10 s of the video. Following the video clip an on-screen instruction prompted participants to move back to the tablet game. Participants were given three practice trials to become familiar with the set-up (two trials with a tablet game duration of 10 s and one with a duration of 30 s). Following this they completed three blocks of trials, one for each of the games. The order of games was counterbalanced across participants and the practice always incorporated the game they would play first. Between each block they were given a break and were able to familiarise themselves with the next game. Each block consisted of 24 trials, 12 trials with a 10-s duration of the secondary task and 12 trials with a 30-s duration. In six trials there was a hazard and in six there was no hazard. Within each block all trials were presented in a random order.

### Results

Data collected from the first experiment were accuracy and response times to the hazards in the driving clips and the horizontal (*x*) and vertical (*y*) positions of each fixation made in the first 1,500 ms of the driving clips. The timeframe was limited based on previous research showing the carry-over effect did not last beyond 2,000 ms (e.g., Thompson et al., 2015, 2021). The *x* and *y* positions were measured in pixels but converted to degrees of visual angle. Spread of search along the horizontal and vertical axis was then calculated as the *SD* of the *x* and *y* fixation positions. Each of the four dependent variables were analysed using a 3 (orientation) × 2 (duration) within-participants ANOVA.

Analysis of accuracy and response times to hazards was conducted for 36 participants due to missing behavioural data from 4 participants. For overall accuracy in the hazard perception task (whether participants correctly identified a clip as having a hazard or having no hazard) there was no effect of orientation, *F*(2, 70) = 0.139, *MSE* = 175.558, *p* *=* .870, *partial* η^2^ = .004. This is possibly not surprising given that the three games did not elicit substantially different search patterns. There was, however, a significant main effect of duration, *F*(1, 35) = 7.968, *MSE* = 78.116, *p* = .008, *partial* η^2^ = .185, with higher accuracy when participants spent longer on the secondary task (means of 71.76% and 68.36% for the 30 and 10 s conditions, respectively). After removing trials in which there were no hazards (so only analysing accuracy to detect that a video did include a hazard) mean accuracy was much higher (97.53%) and the effect of duration disappeared. There was no interaction between orientation and duration, *F*(2, 70) = 0.874, *MSE* = 66.872, *p* *=* .874, *partial* η^2^ = .004.

Participants were instructed to respond to hazards when they became a hazard, rather than responding to something that could potentially become a hazard. However, response times to the hazards in the driving clips showed that participants were correctly responding an average of 4.72 s early. For response time there was no effect of orientation, *F*(2, 70) = 1.213, *MSE* = 4,092,564.34, *p* *=* .303, *partial* η^2^ = .033. Again, there was a significant effect of duration, *F*(1, 35) = 21.596, *MSE* = 672,097.960, *p* *<* .001, *partial* η^2^ = .384, with responses made significantly earlier in the 10-s block than in the 30-s block. There was no interaction between orientation and duration, *F*(2, 70) < 0.001, *MSE* = 1,132,861.32, *p* *=* 1.000, *partial* η^2^ < .001.

The eye-tracker did not calibrate for six participants and a further two participants were categorised as outliers because their data for the two dependent variables of spread of search along the horizontal and vertical axis were more than 2.5 *SD*s from the mean. Analysis of eye movements in the driving clips was therefore conducted on data from 32 participants. As expected, when searching for hazards in videos of driving, the *SD* of the horizontal position of fixations was larger than the *SD* of the vertical position (means of 2.77° and 1.05°, respectively).

For spread of search along the horizontal axis there was no significant effect of orientation, *F*(2, 62) = 0.140, *MSE* = 0.221, *p* *=* .869; no effect of duration, *F*(1, 31) = 1.906, *MSE* = 0.105, *p* *=* .177, *partial* η^2^ = .058, *partial* η^2^ = .005; and no interaction between orientation and duration, *F*(2, 62) = 0.223, *MSE* = 0.158, *p* *=* .801, *partial* η^2^ = .007. The same pattern of results was found for the vertical spread of search with no effect of orientation, *F*(2, 62) = 0.075, *MSE* = 0.066, *p* *=* .928, *partial* η^2^ = .002, no effect of duration, *F*(1, 31) = 0.591, *MSE* = 0.057, *p* *=* .448, *partial* η^2^ = .019, and no interaction, *F*(2, 62) = 0.169, *MSE* = 0.052, *p* *=* .845, *partial* η^2^ = .005.

### Discussion

The aim of Experiment 1 was to measure carry-over of top-down attentional set between two tasks that incorporated different stimuli and demands and were presented to different spatial locations. This would show whether attentional inertia can occur in more dynamic scenarios. Across three blocks of trials participants played a tablet computer game that induced a more horizontal, vertical, or random spread of search and then viewed hazard perception clips. Eye movements were recorded to the driving videos to provide a measure of attention, and these were compared based on the search used in the preceding game.

In direct contrast to previous findings (e.g., Hills et al., 2016, 2018; Thompson & Crundall, 2011) there was no evidence that the allocation of attention in the hazard perception task was influenced by the orientation of stimuli in the games. The spread of search in the driving clips did not vary in relation to the game played within each block of trials, and the amount of time spent playing a game in each trial also had no impact on subsequent attention and search in the videos.

One explanation for this non-significant effect is that the initial task did not prime attentional settings. This may be because participants did not invest sufficient resources into the games to establish a set, so carry-over was less likely (i.e., Leber & Egeth, 2006). Yet past research shows persistence of top-down attentional settings after very brief exposure to an initial task (e.g., Longman et al., 2013) and in the current study participants spent much longer completing the first task before moving to the second. A more likely account is that the top-down attentional settings adopted for completing the different games were not sufficiently distinctive. This is supported by the pilot study which showed that while eye movements to the three games did vary, the “horizontal” game (Solitaire) still required vertical search and the “vertical” game (Tetris) still required horizontal search. That is markedly different to the simple letter search task used in previous studies where stimuli were only located along the horizontal or vertical axis so search would be more constrained and therefore more likely to prime distinctive settings. Consequently, the top-down attentional settings may have persisted from the games to the video clips, but this was not apparent in the vertical and horizontal spread of search. This highlights a limitation with this method; carry-over of attentional set is only being inferred through the measure of eye movements and there is no performance measure that compares the processing of stimuli presented to previously relevant and previously irrelevant locations.

A further key difference between this experiment and the previous research was the requirement for participants to make a spatial shift between the two tasks. In each previous study utilising this paradigm the tasks were presented to the same display, and all experiments demonstrated some form of carry-over. In the present experiment the two tasks were shown to different displays, requiring a participant to reorient their body in space (moving 90° to the right). It may therefore be suggested that changing position has affected attentional set switching. Specifically, presenting the two tasks to different locations may have eliminated the carry-over effect by triggering a change in attentional set. This was investigated further in Experiment 2.

## Experiment 2

The aim of the second experiment was to compare carry-over of attentional set between two unrelated tasks when the tasks were presented to the same display, or to different displays. One option here would be to replicate the method from Experiment 1 but show both tasks (games and hazard perception clips) to the same spatial location. However, while it is proposed that the presentation of the two tasks to different displays played a role in the non-significant carry-over effect, as discussed above there are other factors that may have contributed to the results. To increase the likelihood of a carry-over effect in a static set-up (and therefore allow a comparison of this with a dynamic set-up), the original experimental paradigm of Thompson and Crundall (2011) was used with participants searching through letters arranged horizontally, vertically, or randomly before viewing an image for 2,000 ms. It was predicted that orientation of the letters would influence spread of search in the pictures when both tasks were presented to the same spatial location, but no carry-over effect would be found when the tasks were presented to different spatial locations.

### Method

#### Design

A 3 (orientation) × 2 (screen) within-participants design was used. In the letter search stimuli were arranged in one of three orientations (horizontal, vertical, or random). Participants completed two blocks of trials, one in which the two tasks (letter search and picture search) were presented to the same computer screen (one-screen) and one in which the two tasks were presented to different screens (two-screen). The dependent variables were spread of search along the horizontal and vertical axis in the first 1,000 ms of the picture search, taking the *SD* of the *x* and *y* locations of each fixation (in degrees of visual angle). Accuracy and response times were collected for the letter search task and a complexity rating was collected from each participant for each image in the picture search task. Ethical approval was granted from the Health Sciences Research Ethical Approval Panel at the University of Salford (HSR1819-065).

#### Participants

The same as Experiment 1, based on an a-prior power analysis using an effect size from Thompson et al. (2021), a sample size of 33 participants was required, and in total 40 participants (22 female) completed the experiment for a £10 inconvenience allowance. Age ranged from 18 to 52 and mean age was 25.88. All participants were staff or students from the University of Salford and all reported normal or corrected-to-normal vision (there was no requirement to have a driving licence in this experiment).

#### Stimuli and apparatus

The experiment was designed and run using E-Prime Pro 2 on a Viglen genie computer with an Intel Core i7-6700 processor. The size of both screens used in the experiment was 24-in. (531 mm × 298 mm; aspect ratio 16:9); however, the stimuli were presented to an area measuring 381 mm × 298 mm (with a black border to the left and right edges of the screen). Eye movements in the picture search task were recorded using a Tobii Pro Spectrum with a sampling rate of 120 Hz. The minimum fixation duration was 100 ms and the minimum fixation dispersion threshold was 100 pixels. In the letter search task, 9 letters were presented on the screen: 4 vowels and 5 consonants or 3 vowels and 6 consonants. Letters were presented in Verdana font size 18 (0.95°× 0.95°) in black on a white background, all letters were used except the letter I, and letters were shown in an upright position and could be uppercase or lowercase. In a horizontal search the letters were presented along the centre of the screen (the full letter string subtended 25.36° × 0.95°), in a vertical search the letters were arranged in a line down the centre of the screen (0.95° × 25.36°), and in a random search the letters could be located anywhere within an invisible 9 × 9 grid (with the display subtending a maximum of 25.36° × 25.36°). The images used for the picture search task were 48 road scenes, 48 nature scenes, and 48 fractal images, all were shown in full colour and measured 35.23°× 27.89°. This gave a total of 144 images, and these were the same used by Thompson et al. (2021). When the two tasks were presented to the same screen, they were presented on the Tobii Pro Spectrum. When the two tasks were presented to different screens (two-screen block) the picture search was presented on the Tobii Pro Spectrum and the letter search was presented on a monitor situated on a desk 90° to the left of the eye-tracker. Two chin rests were used, one in front of each screen and these ensured participants were 60 cm from the screen when completing the experiment.

#### Procedure

Participants were given full instructions and provided written informed consent. They completed two blocks of trials with a break between each block. In the one-screen block eye movements were calibrated using a 5-point calibration screen. In each trial, a fixation cross was presented to the centre of the screen in black on a white background for 500 ms. A letter search was then presented, and participants were instructed to count the number of vowels and respond by pressing the numbers “3” or “4” on the keyboard. They were given feedback via a green or red blank screen that was presented for 1,000 ms. In 50% of the trials another two letter searches were presented. The letters were different each time but within a given trial they were always presented in the same orientation, and again, feedback was provided for 1,000 ms after each response. An image was then shown for 2,000 ms. Participants were asked to view this image and when it disappeared on-screen instructions asked them to “please rate the complexity of the image you have just seen on a scale from 1 (*low complexity*) to 6 (*high complexity*).” On completion of this task there was an intertrial interval of 1,500 ms featuring a blank white screen and then the next trial began with the fixation cross. The procedure was the same for the two-screen block with the exception that following calibration of eyes participants were given on-screen instructions to “please now move to the left-hand screen for the letter search task” at which point they had to move to the computer to their left, place their chin in the chin rest and complete the letter search. The instructions to move screen were presented for 1,500 ms (matching the intertrial interval in the one-screen block). After either one or three searches, participants were presented with the feedback display for 1,000 ms and after 500 ms (i.e., mid-way through the feedback) a tone (ElectronicChimeSound.mp3) sounded for 500 ms at which point they had to move to the eye-tracking computer, place their chin in the chin rest and complete the picture search. The time between the letter search and the picture search was therefore consistent (1,000 ms) for the one-screen and two-screen blocks. After providing the complexity rating the on-screen instructions reminding them to move back to the left-hand computer for another trial were again presented for 1,500 ms. Participants completed 72 trials in each block, 24 for each condition of orientation. Within these 24 trials, there were 12 trials with one letter search and 12 trials with three letter searches, with four trials for each image type (roads, nature images, fractals). There were an equal number of trials with three or four vowels and all trials were presented in a random order. Block order was counterbalanced across participants.

### Results

A calibration issue for 2 participants meant that data from only 38 participants was analysed. The dependent variables were the *SD* of the *x* and *y* positions of fixations to the images, providing a measure of the horizontal and vertical spread of search. These were analysed using two 3 (orientation) × 2 (screen) within-participants ANOVAs. Any effect of orientation was further analysed using planned comparisons that compared the horizontal and vertical conditions with the random condition. Prior to analysis all trials in which participants responded inaccurately to the letter search immediately preceding the image were removed (7.62% of all trials).

Each image was presented for 2,000 ms; however, analysis was only conducted for fixations in the first 1,000 ms based on the findings from Thompson et al. (2015). For trials in the one-screen block the first fixation made on the images was removed to ensure that any carry-over did not reflect the eyes moving back to the expected position of the first letter of the preceding letter search task. Given that participants completed each task on different screens in the two-screen block, and to accommodate the time taken to move screen, the first fixation was not removed from trials in the two-screen block.^1^

**Figure 3.**
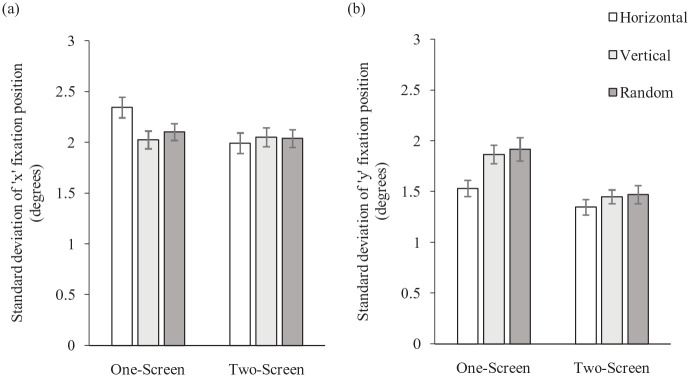
Showing the mean spread of search along the (a) horizontal and (b) vertical axes in the images in Experiment 2. While search varied according to orientation of stimuli in the letter search task when the full experiment was presented to one screen, these differences were no longer found when the letter task and the image task were presented to two separate screens. Error bars represent standard error.

For spread of search along the horizontal axis (see Figure 3a) there was a significant effect of orientation, *F*(2, 74) = 4.057, *MSE* = 0.087, *p* *=* .021, *partial* η^2^ = .099. The planned comparisons showed significantly wider horizontal search following horizontally presented letters (*M* = 2.17°) compared with random (*M* *=* 2.07°), *F*(1, 37) = 4.413, *MSE* = 0.166, *p* *=* .043, *partial* η^2^ = .107. There was no significant difference in horizontal search following vertical (*M* = 2.04°) and random letters, *F*(1, 37) = 0.494, *MSE* = 0.164, *p* *=* .487, *partial* η^2^ = .013. There was a non-significant effect of screen, *F*(1, 37) = 3.102, *MSE* = 0.314, *p* *=* .086, *partial* η^2^ = .077; however, there was a significant interaction between orientation and screen, *F*(2, 74) = 5.387, *MSE* = 0.137, *p* *=* .007, *partial* η^2^ = .127. Again, this was found between the horizontal and random conditions, *F*(1, 37) = 4.517, *MSE* = 0.346, *p* *=* .040, *partial* η^2^ = .109, with no difference between the vertical and random conditions, *F*(1, 37) = 0.628, *MSE* = 0.244, *p* *=* .433, *partial* η^2^ = .017. This showed that while horizontal spread of search was greater following horizontal letters than random letters in the one-screen block (means of 2.34° and 2.10°, respectively), this difference was not found in the two-screen block, with a mean of 1.99° for the horizontal condition and 2.04° for the random condition.

For spread of search along the vertical axis (see Figure 3b) there was a significant effect of orientation, *F*(2, 74) = 11.236, *MSE* = 0.129, *p* *<* .001, *partial* η^2^ = .233. This was again driven by the difference between the horizontal and random letter conditions, *F*(1, 37) = 18.806, *MSE* = 0.263, *p* *<* .001, *partial* η^2^ = .337, as participants showed significantly wider vertical search following randomly presented letters (*M* = 1.69°) than following horizontal letters (*M* = 1.44°). There was no difference in vertical search following vertical (*M* = 1.66°) and random letters, *F*(1, 37) = 0.436, *MSE* = 0.287, *p* *=* .560, *partial* η^2^ = .009. There was a significant effect of screen, *F*(1, 37) = 10.793, *MSE* = 0.644, *p* *=* .002, *partial* η^2^ = .226, as participants searched more widely along the vertical in the one-screen set-up (*M* = 1.77°) than the two-screen set-up (*M* = 1.42°). Crucially, there was also a significant interaction between orientation and screen, *F*(2, 74) = 3.807, *MSE* = 0.102, *p* *=* .027, *partial* η^2^ = .093. While vertical search was wider following random (and vertical) letters compared with horizontal, *F*(1, 37) = 6.777, *MSE* = 0.190, *p* *=* .013, *partial* η^2^ = .155, this was only for the one screen block with means of 1.53° (horizontal), 1.87° (vertical), and 1.92° (random). In the two-screen block mean vertical search was similar across the horizontal (1.35°), vertical (1.45°), and random conditions (1.47°). There was no significant interaction between orientation and screen for the vertical and random conditions, *F*(1, 37) = 0.060, *MSE* = 0.264, *p* *=* .807, *partial* η^2^ = .002.

Accuracy and response times to the letter search task was not of primary interest in this experiment, but a separate analysis on this task showed that accuracy did not vary across the three conditions of orientation, *F*(2, 74) = 0.213, *MSE* = 66.410, *p* *=* .808, *partial* η^2^ = .006, and did not vary according to whether the experiment was completed on one screen or two screens, *F*(1, 37) = 0.419, *MSE* = 265.269, *p* *=* .552, *partial* η^2^ = .011. There was also no interaction between orientation and screen, *F*(2, 74) = 0.344, *MSE* = 49.129, *p* *=* .710, *partial* η^2^ = .009. Analysis of response times showed a significant effect of orientation, *F*(2, 74) = 25.577, *MSE* = 133,026.703, *p* *<* .001, *partial* η^2^ = .409. Participants were significantly quicker to accurately identify whether 3 or 4 vowels were presented when the letters were shown horizontally compared with when shown randomly across the screen (means of 2,641 and 2,887 ms, respectively), *F*(1, 37) = 38.166, *MSE* = 242,561.004, *p* *<* .001, *partial* η^2^ = .508. There was no difference in response times to random and vertical letters (mean RT of 2,911 ms), *F*(1, 37) = 0.225, *MSE* = 361,944.390, *p* *=* .638, *partial* η^2^ = .006. Response times for the letter search task did not vary significantly across the two blocks (one screen or two screen), *F*(1, 37) = 3.623, *MSE* = 378,183.991, *p* *=* .065, *partial* η^2^ = .089; however, this was approaching significance with response times marginally quicker in the two-screen block (*M* = 2,729 ms) compared with the one screen block (*M* = 2,896 ms). There was no interaction between orientation and screen, *F*(2, 74) = 0.112, *MSE* = 93,834.186, *p* *=* .894, *partial* η^2^ = .003.

When viewing the images participants were asked to rate them for complexity. Again, the ratings given were not of primary interest; however, these were analysed using a 3 (orientation) × 2 (screen) × 3 (image type) within-participants ANOVA. This showed that complexity ratings did not differ due to the orientation of the letter search, *F*(2, 74) = 2.717, *MSE* = 0.122, *p* *=* .073, *partial* η^2^ = .068, and did not differ when completing the experiment on one screen or two screens, *F*(1, 37) = 0.925, *MSE* = 0.699, *p* *=* .342, *partial* η^2^ = .024. Ratings were, however, affected by the image type, *F*(2, 74) = 66.160, *MSE* = 3.751, *p* *<* .001, *partial* η^2^ = .641. Bonferroni-corrected pairwise comparisons showed significantly higher complexity ratings for the fractal images (*M* = 4.93) compared with the nature (*M* = 3.03; *p* *<* .001) and road images (*M* = 3.23; *p* *>* .001), but complexity ratings did not differ significantly for nature and road images (*p* = .648). This was consistent with the findings of Thompson et al. (2021).

### Discussion

Experiment 2 aimed to compare carry-over of attentional settings between two tasks when those tasks were presented to the same, or different spatial locations. Adopting the more artificial experimental paradigm used by Thompson and Crundall (2011), participants searched through horizontal, vertical, or random letter arrays and then viewed an image for 2,000 ms. The letter search and the picture search were presented to the same display or to different displays and the results showed that when presented to two different spatial locations there was no carry-over of attentional settings between the two tasks and the horizontal and vertical spread of search in the picture task was not affected by the orientation of the preceding letter search. In contrast, when the tasks were shown to the same display, the orientation of letters did influence search in the picture task; horizontal spread of search was wider in the pictures following horizontally presented letters compared with randomly presented letters, and vertical spread of search was narrower following horizontally presented letters. The findings from the one-screen block are consistent with the findings of Thompson et al. (2021) who used the same letter search task and the same images in the picture search task. Given that the two experimental blocks were identical aside from the spatial shift between the tasks in the two-screen block, the results suggest that persistence of attentional settings will not occur in dynamic settings that involve directing attention to different tasks presented to different areas of space.

Although the key difference between the two conditions in Experiment 2 was the spatial shift required between the letter search and picture search tasks, the nature of the methodology raises two alternative explanations for the findings. The first is that the time needed to switch between two displays in the two-screen block provided sufficient time for a set switch. Longman et al. (2013, 2017); have found that when participants have more time to prepare for a switch attentional inertia disappears. In this experiment, although the onset of the picture was 1,000 ms after the immediately preceding letter search task in both the one-screen and two-screen blocks, there is no way to assess whether participants took longer to engage their attention on the pictures in the two-screen block. If they did then this would allow greater preparation time, reducing any carry-over from the letter search. In relation to task switching, Rogers and Monsell (1995) argued that while one can prepare for a task switch, the set will not be fully reconfigured until the stimuli associated with the set are presented because they exogenously trigger the set. This may be one reason why Longman and colleagues did not find attentional inertia when the interval between two tasks increased, but still found evidence of performance costs associated with set switching. Again, there is no way to assess this with the current experimental paradigm because the “costs” associated with set switching are only inferred through the carry-over of visual search and no measure of performance was taken.

Even if the time taken to attend to the pictures was the same in both blocks, they were still not comparable in that the two-screen block effectively involves an additional task between the letter and picture search; that of moving from one chin rest to another. Therefore, any carry-over from the letter search may be observed during this movement and so may no longer be present when participants fixated the pictures. Indeed, if there is a carry-over of attentional settings it could even be argued that the settings most likely to persist to the picture search (in the two-screen block) are those associated with locating the chin rest.

Both the potential delay and the additional task prior to the set switch when two tasks are shown to different displays would account for the findings in Experiment 2, and those in Experiment 1. To improve upon the current design and potentially rule out these explanations, it would be useful to vary the inter-trial interval between the letters and pictures in the one-screen block. It would also be beneficial to measure attention throughout the whole experiment (e.g., by using eye-tracking glasses or EEG) or add a comparable task with the one-screen block.

## General discussion

There is a growing body of evidence to show that the top-down attentional set adopted to complete one task can persist to a second task, influencing attention, visual search, and performance. This has been demonstrated in low-level tasks measuring the bias of attention towards previously relevant information (e.g., Leber & Egeth, 2006; Thompson et al., 2007) and to previously relevant areas of space (e.g., Hills et al., 2017; Longman et al., 2013; 2017; Thompson & Crundall, 2011; Thompson et al., 2021; Wendt et al., 2017). Longman et al. (2013, 2017) argue that the carry-over of attentional set (which they refer to as attentional inertia) occurs because a set switch requires adoption of the new attentional settings and inhibition of the old settings. Until the old settings can be inhibited, they will continue to influence attention in a new task, even when irrelevant. This is supported by findings of Hills et al. (2016) that the carry-over effect is larger when participants over-orient attention in an initial task, as this leads to greater difficulties disengaging from previously relevant stimuli and spatial locations and so more difficulty inhibiting the old set.

Despite the evidence for attentional inertia, there is an argument that the past research utilises experimental paradigms that do not reflect dynamic environments. For example, in the paradigm developed by Thompson and Crundall (2011), carry-over is measured from a simple letter search task to a picture or video search, with both tasks presented to the same display. The aim of the current work was to explore whether attentional inertia has an impact in more dynamic scenarios. In Experiment 1, participants played a computer game using a tablet device and then searched through hazard perception clips. Three computer games were selected that encouraged a more horizontal, vertical, or random spread of search, although they did not constrain attention to the same extent as the letter searches used in previous research. The switch between the two tasks also incorporated a spatial shift as the hazard perception clips were presented to a monitor 90° to the right of the tablet device. The results showed no indication that the characteristics of the games influenced subsequent attention in the driving clips (based on a comparison of spread of search across the three conditions), and in contrast to previous findings (Hills et al., 2018; Thompson & Crundall, 2011) the way in which attention was allocated in the first task did not affect identification of hazards.

To explore whether the spatial shift between the two tasks had any impact on set switching (and therefore the carry-over of attentional settings), Experiment 2 returned to the basic paradigm of presenting letters (oriented horizontally, vertically, and randomly) and then asking participants to search a picture either presented to the same display, or a different display. When the two tasks were shown to the same display, there was a carry-over effect with a wider horizontal spread of search and a narrower vertical spread of search after viewing horizontally presented letters. While this aligned with previous findings using this paradigm, when the tasks were shown to different displays there was no carry-over effect.

One explanation for the difference when tasks are presented to one or two displays is that individuals adopt different strategies to allocate visuospatial attention according to the demands of the situation (e.g., Leber & Irons, 2019). An example relevant to the current work is the strategy for allocating visuospatial attention. According to Galati et al. (2010), there are many ways in which an individual can allocate visuospatial attention, although research focuses on two key reference frames: viewer-dependent and environment-dependent. A viewer-dependent visuospatial frame of reference means processing external information in relation to oneself (e.g., “the chair is in front of me”) and an environment-dependent visuospatial frame of reference means processing external information in relation to other information (e.g., “the chair is next to the table”). A viewer-dependent spatial frame of reference is favoured in smaller, static environments, and an environment-dependent frame is most useful in dynamic environments (Jiang & Swallow, 2013). Given the findings presented here, it may be suggested that when two tasks are presented to the same area of space (i.e., a static environment), information is processed relative to the observer and when a task changes the attentional settings will persist (as they remain with the observer). In a more dynamic set-up when two tasks are presented to different spatial locations, attention may be allocated using an environment-dependent reference frame; therefore, any persistence of previously relevant settings will remain with the environment and will have no impact on attention and search in the new task.

The biasing of attention to previously relevant locations due to the use of a viewer-centred spatial frame of reference is supported by the research of Jiang and Swallow (2013). Across three experiments, participants were asked to search for a target (*T*) among distracters (*L*s) on a display presented flat on a table (facing upwards). In a training phase of trials, the target was more likely to be located in a specific quadrant of the display, although participants were not informed of this. Over the training phase, there was evidence that participants were adopting an attentional bias to the target-rich quadrant, representing incidentally learned attention. Jiang and Swallow also found that the attentional bias persisted to a testing phase when probability of the target location was equal across all quadrants. However, between the training and testing phases participants moved 90° around the display and it was found that the bias remained with the participant, also moving 90°. This means that attention was allocated from a viewer-centred frame of reference and this frame of reference did not update when the participant moved around the display. These findings seem to contrast with the present results showing that a movement of 90° led to an update in top-down attentional settings. However, Jiang and Swallow asked participants to complete a large number of training trials, and while the participant moved, the display remained the same. In the current experiments the two tasks participants complete within each trial are substantially different, and they spend a limited amount of time on each task.

In a further study using a large outdoor environment, Jiang et al. (2014) again found evidence of incidental learning guiding the allocation of attention, but they also found that attention could be allocated either from a viewer-centred or an environment-centred frame of reference. This led them to argue that different forms of attention may incorporate different reference frames. In particular, they associated attentional shifting with greater flexibility in the frame of reference. Based on the current findings, it may be proposed that a viewer-centred frame of reference limits attentional shifting, leading to the persistence of settings between two tasks. This may indicate that the carry-over of attentional settings will only occur in static tasks and so may not pose an issue in dynamic situations. This is consistent with the conclusion of Lien et al. (2010) that attentional settings can be reconfigured quickly and effectively in dynamic conditions.

It is proposed that when orienting attention to a single display an observer will show preference for a viewer-centred frame of reference, and when orienting attention to different areas of space they will be biased towards an environment-centred frame of reference. With a viewer-centred strategy, any attentional weights that bias resources to specific features and locations will remain with the observer when the task changes, therefore affecting subsequent attention. With an environment-centred strategy, persistence of attentional settings will not occur because the weightings remain with the environment and new attentional settings will be adopted as task demands change. This fits with the spatial scale hypothesis of Jiang and Won (2015) that a small-scale, “figural” space (the size of a picture, or a computer screen), will encourage a viewer-centred frame of reference, but a large-scale, “vista” space, will lead to an environment-centred frame of reference.

However, in the present work it is impossible to know whether participants were indeed making use of different reference frames or attentional strategies. To provide additional support for this explanation, it would be useful to either measure the spatial reference frame used in each block or to explicitly ask participants to adopt a specific strategy and measure the effect of this on carry-over. Leber and Irons (2019) have outlined a range of techniques for measuring the effects of different attentional strategies. They also report findings showing that individuals can choose different attentional control settings in more dynamic tasks (Irons & Leber, 2016). Research from Chiu et al. (2020) has also shown that performance costs associated with task switching can be reduced when participants are primed (bottom-up) to voluntarily switch tasks. There is clear evidence for the adoption of different attentional strategies according to different situational demands, but this would need to be investigated further to determine if it has affected the current results.

The two experiments presented here add an important contribution to the research surrounding attentional inertia, and by studying switching under more dynamic settings the work may also contribute to theoretical explanations regarding the activation of attentional settings. More traditional accounts of set switching argue that the allocation of attention to relevant information is achieved through top-down control. The costs of switching tasks occur due to the time taken to reconfigure settings for the new task (task set reconfiguration account; e.g., Rogers & Monsell, 1995) or the difficulty inhibiting previously relevant settings from the old task (task set inertia account; e.g., Allport et al., 1994). More recent explanations have proposed that attentional control settings can be activated in a bottom-up manner (e.g., Dignath et al., 2019). For instance, King et al. (2012) proposed that contextual cues prime “the rapid (and implicit) retrieval and implementation of particular top-down control settings” (p. 8192). They measured this by asking participants to complete a flanker task in which the flanker display was presented to the left or right of the screen. The proportion of trials in which the flankers were congruent and incongruent to the target was manipulated and aligned to either the left or the right: for example, when shown to the right 75% of the trials were congruent and 25% were incongruent, when shown to the left 25% of the trials were congruent and 75% were incongruent. Results showed that the ability to inhibit distracters varied across the different contexts (locations), despite participants having no explicit awareness of the manipulation. The findings support the priming of control hypothesis (e.g., Snapé & Hommel, 2008; Verguts & Notebaert, 2008, 2009) and King et al. presented the argument that top-down control settings may be stored alongside contextual information in episodic representations. Thus, when the event file is triggered by context, the other information bound within the representation (the attentional settings) is also triggered.

The current findings seem consistent with the argument that context can prime the activation of attentional control settings, because it would be presumed that a greater contextual change would act as a more effective prime. It would therefore be predicted that if two tasks share the same stimuli (regardless of the relevance of the stimuli) or are presented to the same spatial location (e.g., Leber & Egeth, 2006; Wendt et al., 2017), they would trigger the same attentional settings, and therefore, there would be a greater chance that attentional settings persist from one task to another because there are limited cues to update the attentional settings. When there is greater contextual variation between two tasks (i.e., in the two experiments presented here), there will be no carry-over because the change in context exogenously triggers a change in attentional settings, leading to more effective set switching. This would need to be investigated in a systematic manner and rather than selecting two tasks that differ substantially from the outset (as in Experiment 1), it may be prudent to use tasks that are highly similar and measure the persistence of attentional settings in conjunction with gradual increases in contextual variation.

The main aim of the present work was to explore whether persistence of top-down attentional settings from a task in which they are relevant to a task in which they are no longer relevant would affect the allocation of resources in dynamic settings. Two experiments measured how the orientation of attention in an initial task influenced spread of search in a second task when the two tasks incorporated different demands, participants spent limited time completing each task, and the tasks were shown to different spatial locations. The findings indicate that attentional inertia will be more costly in static conditions and will have limited impact on the allocation of attention in dynamic situations. Based on previous findings, it may be speculated that the increased contextual variation found in dynamic settings primes a reconfiguration of settings or primes the adoption of a more flexible attentional strategy.

## Notes

1 Additional analysis of the data with the first fixation also removed from the two-screen block revealed the same pattern of results as presented here. Removing this first fixation from the two-screen block did, however, lead to fewer fixations in this additional analysis compared with the one-screen block, reflecting the difficulties in the eye-tracker finding the eyes of the participants after the change in tasks. Future research would benefit from using eye-tracking glasses to allow for constant recording across both tasks.
